# Nuclear conditions of basidiospores and hyphal cells in the edible mushroom *Oudemansiella aparlosarca*

**DOI:** 10.1002/mbo3.1233

**Published:** 2021-09-26

**Authors:** Roy Rebecca, Qi Gao, Yujin Cui, Chengbo Rong, Yu Liu, Wensheng Zhao, Wasantha Kumara, Shouxian Wang

**Affiliations:** ^1^ Key Laboratory of Urban Agriculture (North) Institute of Plant Protection Beijing Academy of Agriculture and Forestry Sciences Beijing Engineering Research Center for Edible Mushroom Ministry of Agriculture Beijing China; ^2^ Department of Agricultural Biology Faculty of Agriculture University of Ruhuna Kamburupitiya Sri Lanka; ^3^ China Agricultural University Beijing China

**Keywords:** amphithallic life cycle, binucleate, multinucleate, *Oudemansiella aparlosarca*, post‐meiotic mitosis, spore size

## Abstract

*Oudemansiella aparlosarca* is an edible mushroom possessing medicinal and health benefits. Although there are studies on the cultivation of *O*. *aparlosarca*, only a few studies have focused on its genetics and life cycle. Therefore, the main objective of this study was to identify the nuclear conditions of basidiospores and homokaryotic and heterokaryotic hyphal cells and to determine the influence of different nuclear conditions on basidiospore diameter in *O*. *aparlosarca*. Two parental strains: strain‐55 and strain‐81 were used. Staining of basidiospores and hyphal cells in the apical region was performed. We observed the following nuclear conditions: non‐nucleate, mononucleate, binucleate, and multinucleate. In both parental strains, binucleate spores were predominant, while the number of non‐nucleate spores was the lowest. The diameter of non‐nucleate spores was the smallest, being 11.52 µm and 12.15 µm in parental strain‐81 and strain‐55, respectively, while multinucleate spores had the largest diameter, being 14.78 µm in both parental strains. Both homokaryotic and heterokaryotic strains were identified in isolated single spores from parental strains. Binucleate cells were majorly present in heterokaryotic hyphal cells, and multinucleate cells were predominant in homokaryotic hyphal cells. We conclude that *O*. *aparlosarca* contains homokaryotic and heterokaryotic basidiospores, which indicates an amphithallic life cycle. The observed binucleate spores might be the result of post‐meiotic mitosis.

## Graphical abstract

The life cycle of the fungus *Oudemansiella aparlosarca* includes homokaryotic and heterokaryotic basidiospores with four nuclear conditions: non‐nucleate, mononucleate, binucleate, and multinucleate. The number of binucleate cells was high in heterokaryotic hyphal cells, while that of multinucleated cells was high in homokaryotic hyphal cells. The predominance of binucleate spores might be a result of post‐meiotic mitosis.

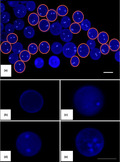

## 1 INTRODUCTION

*Oudemansiella aparlosarca*, belonging to the family Physalacriaceae, is a ubiquitous species of edible mushrooms (Kirk et al., 2008; Magingo et al., 2004). *Oudemansiella* species exert anti‐cancer, antihypertensive (Tsantrizos et al., 1995), antimicrobial, and antibacterial (Rosa et al., 2005) effects and contain bioactive compounds (Liu et al., 2013), such as oudemansin (Anke et al., 1983), oudenone (Tsantrizos et al., 1999), lectin (Liu et al., 2013), mucidin (Šubík et al., 1974), and polysaccharides (Zou, 2005). Oudenone is especially used to treat hypertensive diseases (Tsantrizos et al., 1995).

To enhance the production and quality of edible mushrooms, several genetic studies have been conducted on mushrooms to optimize strains and breeding strategies (Kothe, 2001). Although efforts to cultivate *O*. *aparlosarca* have been successful, a limited number of morphological and nuclear genetic studies have been conducted on basidiospores and homokaryotic and heterokaryotic hyphae from this species (Corner, 1948; Webster & Weber, 2007). Additionally, few studies have been conducted on their nuclear condition; hence, adequate information on the genetics and life cycle of *Oudemansiella* is not available.

In general, the following types of sexual life cycles are observed in fungi: (1) Heterothallic life cycle, which gives rise to self‐sterile homokaryotic hyphae *via* tetrasporic basidia. Plasmogamy between two sexually compatible homokaryons restores fertile heterokaryons (Callac et al., 1993; Kerrigan et al., 1994; Ling et al., 2019; Raper, 1976). (2) Homothallic life cycle, which gives rise to fertile heterokaryotic hyphae that have the genetic capability to form fruiting bodies through auto fertilization (Callac et al., 1998; Dias & Brito, 2017; Kerrigan et al., 1999). Different types of the homothallic life cycle can be observed based on the number of nuclei and nuclear migration. Primary homothallism originates from a self‐fertile mononuclear single spore, and the fungus can complete its life cycle and produce fruiting bodies. Secondary homothallism, also known as pseudo‐homothallism, is mostly observed in bisporic basidia, wherein two post‐meiotic nuclei carrying different mating alleles migrate to the same spore, giving rise to heterokaryotic fertile hyphae (Callac et al., 1993; Dias & Brito, 2017; Kerrigan et al., 1993; Ling et al., 2019; Raper et al., 1972; Sonnenberg et al., 2016, 2017). However, the basidiospores produced *via* secondary homothallism are not always auto fertile heterokaryons, and they are often accompanied by a heterothallic life cycle. (3) Amphithallism, where spores produced by the same basidiocarp follow both heterothallic (homokaryotic spores) and secondary homothallic (heterokaryotic spores) life cycles (Callac et al., 1996; Dias & Brito, 2017; Sonnenberg et al., 2016, 2017). Amphithallism has been reported in more than 10 different basidiomycetes, including *Agaricus brasiliensis*, *A*. *bisporus*, and *Volvariella volvacea* (Baumgartner et al., 2012; Callac et al., 1996; Chen et al., 2016; Ling et al., 2019; Thongklang et al., 2014).

Nucleus‐based studies are necessary to elucidate the life cycle of mushrooms. Therefore, we conducted this study to describe the different nuclear conditions in basidiospores and homokaryotic and heterokaryotic hyphal cells, and to determine the influence of different nuclear conditions on the diameter of basidiospores of two parental strains of *O*. *aparlosarca*. We expect that the findings of this study will support future breeding and genetic studies as well as the production of quality mushrooms from *Oudemansiella* species.

## 2 MATERIALS AND METHODS

### 2.1 Strains, culture maintenance, and fruiting body production

Two parental strains of *O*. *aparlosarca* were used: JZB2115055 (named as strain‐55) and JZB2115081 (named as strain‐81). These two parental strains were identified by Dr. Hao Yanjia of the Kunming Institute of Botany, Chinese Academy of Sciences, following the method reported by Hao et al. (2016). Strains 55 and 81 were collected from Dadugang, Yunnan Province, China, in 2011, and Baiyun Mountain, Guangdong Province, China, in 2013, respectively, and were preserved at the Beijing Engineering Research Center for Edible Mushroom, Beijing Academy of Agriculture and Forestry Sciences, Beijing, China. In 2016, our previously reported strain, *O*. *canarii* (Xu et al., 2016), was renamed as *O*. *aparlosarca* by the Key Laboratory for Plant Diversity and Biogeography of East Asia, Kunming Institute of Botany, Chinese Academy of Sciences. The strains were cultured on potato dextrose agar (PDA) medium at 25°C. The substrate consisted of 50% cottonseed hull, 30% sawdust, 18% wheat bran, and 2% lime, and had a moisture content of 62% (w/w). One kilogram of the substrate was put in a polyethylene bag (16 cm × 32 cm × 0.04 cm) and autoclaved at 121°C for 120 min. The sterilized substrate was inoculated by spreading the spawn on its surface at a concentration of 2% (w/w) fresh weight of the substrate. The bags were kept in a spawn running room at 25°C and 70% relative humidity (RH) under dark conditions. After completion of mycelium running, the bags were unfolded to induce fruiting body development and then maintained at 22°C and 85% RH. Fruiting bodies were harvested at the same mature stage when mushroom caps were open.

### 2.2 Spore suspension and single spore isolation

Six plastic bottles, each containing 100 ml of distilled water, were autoclaved at 121°C for 30 min. Mature fruiting bodies of both parental strains were harvested using a sterile knife, and the mushroom surface was sterilized using cotton soaked in 75% alcohol under sterile conditions in a laminar flow cabinet. The stipe of the mushroom was shortened using a sterilized blade, and it was hung on a hook, which was then fixed to an autoclaved bottle lid, with the mushroom gills facing downwards. These procedures were performed in a laminar flow cabinet. Spore suspensions were collected after two days. One part of the suspension was used for staining, and the other part was subjected to sequential dilution with sterilized water to obtain a concentration of approximately 1 × 10^3^ spores/ml. One hundred microliters of the spore suspensions was spread onto PDA plates. After 24 h, single spores were immediately picked up using a fine sterile needle under a surface light microscope (Olympus stereo microscope SXZ10; Olympus, Tokyo, Japan), and incubated at 25°C on PDA plates for germination. The percentage of germination was calculated. A polymerase chain reaction (PCR) of the internal transcribed spacer (ITS) region was performed to confirm species identity. DNA was extracted from 50 mg of hyphal samples using Plant Genomic DNA Kit (TIANGEN) according to the manufacturer's general protocol. A single‐step 35 cycle PCR using the 2×TSINGKE Master Mix (blue) kit (Tsingke, China) was performed using the primers ITS4 and ITS5 (Rong et al., 2015).

The sequences of the renamed parental strains (this study) and the isolated single spore strains (hereafter, referred to as progeny, originating from a single spore) were checked with the original sequence of *O*. *canarii*, which is available in the NCBI GenBank database under the accession number MK336783.1.

### 2.3 Spore and hyphal cell staining

Fresh spore suspensions were immediately collected from parental strains to observe the different nuclear conditions of the basidiospores of *O*. *aparlosarca*. The suspension (50 μl) was placed on a microscope glass slide and heated to 50°C until all the water evaporated. It was immediately stained with a solution consisting of a 1:1 ratio of 4 PPM 4′, 6‐diamidino‐2‐phenylindole (DAPI; Sigma‐Aldrich, St. Louis, MO, USA) and 2 PPM calcofluor white (Sigma‐Aldrich). The glass slide was covered with an alcohol‐cleaned coverslip, and the edges were sealed with transparent nail polish. To enhance stain penetration, the prepared slides were heated at 50°C for 10 s and immediately cooled inside an icebox for 1 min. Again, the slides were heated at 50°C for 30 s and cooled for 1 min; this procedure was repeated three times. Finally, the stained slides were observed under a UV fluorescence microscope (Olympus IX70 Multi‐parameter Fluorescence Microscope; Olympus, Tokyo, Japan). The experiment was repeated five times, and 2500 spores were counted for each parental strain to observe different nuclear conditions in spores. Additionally, to measure the spore diameter of each nuclear condition, 1000 spores from both parental strains were observed. To categorize the different nuclear conditions in homokaryotic and heterokaryotic hyphal cells in the apical region, the slide culture method was performed as described by Sawada et al. (2014). Briefly, the medium for slide culture was prepared with 1% agar and 0.2% glucose. Petri dishes, forceps, and microscope slides were sterilized. The prepared medium was poured into a small beaker, and sterilized slides were partially dipped into the medium using forceps and kept inside the Petri dishes. Once the medium dried on the slides, a small piece of hypha was placed on each slide and allowed to grow on one half of the slide. Thereafter, the slides were carefully removed from the Petri dishes and gently washed using 0.1 M phosphate‐buffered saline. Hyphae were fixed in an ethanol:acetic acid (3:1, v:v) solution for 20 min at 25°C, stained with a solution containing 4 PPM DAPI (Sigma‐Aldrich) and 2 PPM calcofluor white (Sigma‐Aldrich) in a 1:1 ratio, and observed under a fluorescence microscope (Olympus IX70 Multi‐parameter Fluorescence Microscope; Olympus). A total of 50 hyphal cells in the apical region were manually counted on each culture slide, for both homo‐ and heterokaryotic hyphae. This procedure was repeated thrice. All hyphal staining experiments were performed on the ninth day of culture.

### 2.4 Data analysis

A counting instrument was used to count the number of nuclei in spores and hyphal cells. ImageJ software (National Institutes of Health, MD, United States) was used to measure the diameter of spores and for image analysis. Mean values within the nuclear conditions in a parental strain were subjected to one‐way ANOVA, and significant differences were analyzed by Duncan's multiple range test at 95% confidence level, using the IBM SPSS Statistics software v25.0. Comparisons and significant differences with respect to each nuclear condition between the parental strains were analyzed using the paired‐sample *t* test at a 95% confidence level.

## 3 RESULTS

**FIGURE 1:**
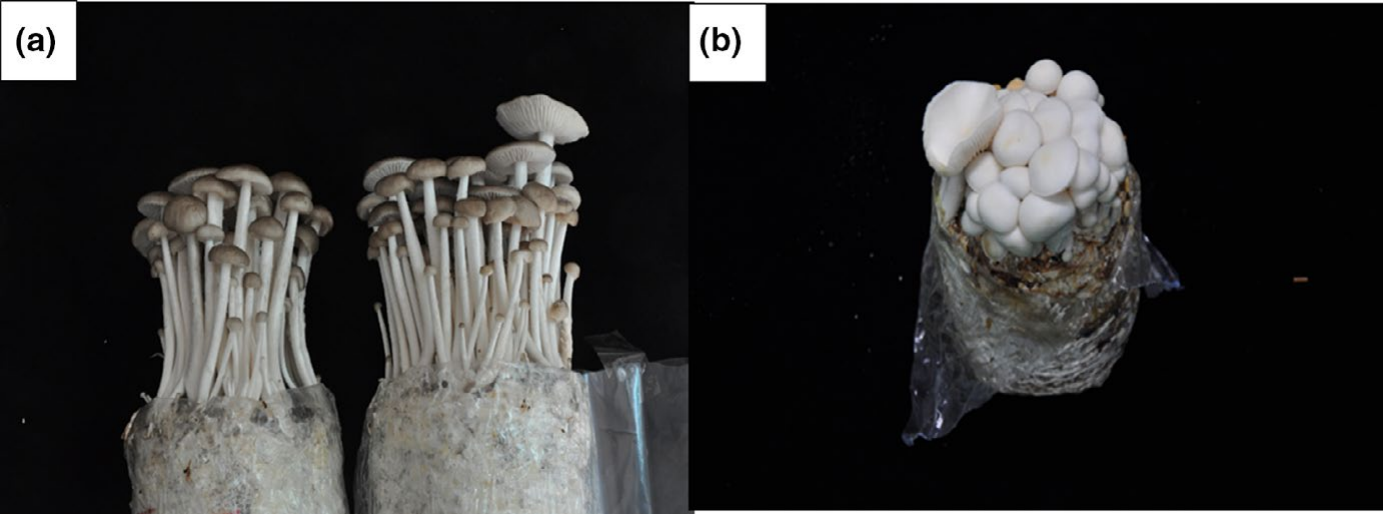
Mature fruiting bodies of (a) Parental strain‐55 and (b) Parental strain‐81

Mature fruiting bodies of both parental strains are shown in Figure 1.

The sequence of parental strains and progeny were highly similar (around 97%–99%) to the original sequence of *O*. *canarii* in the NCBI.

### 3.1 Nuclear conditions of basidiospores

**FIGURE 2:**
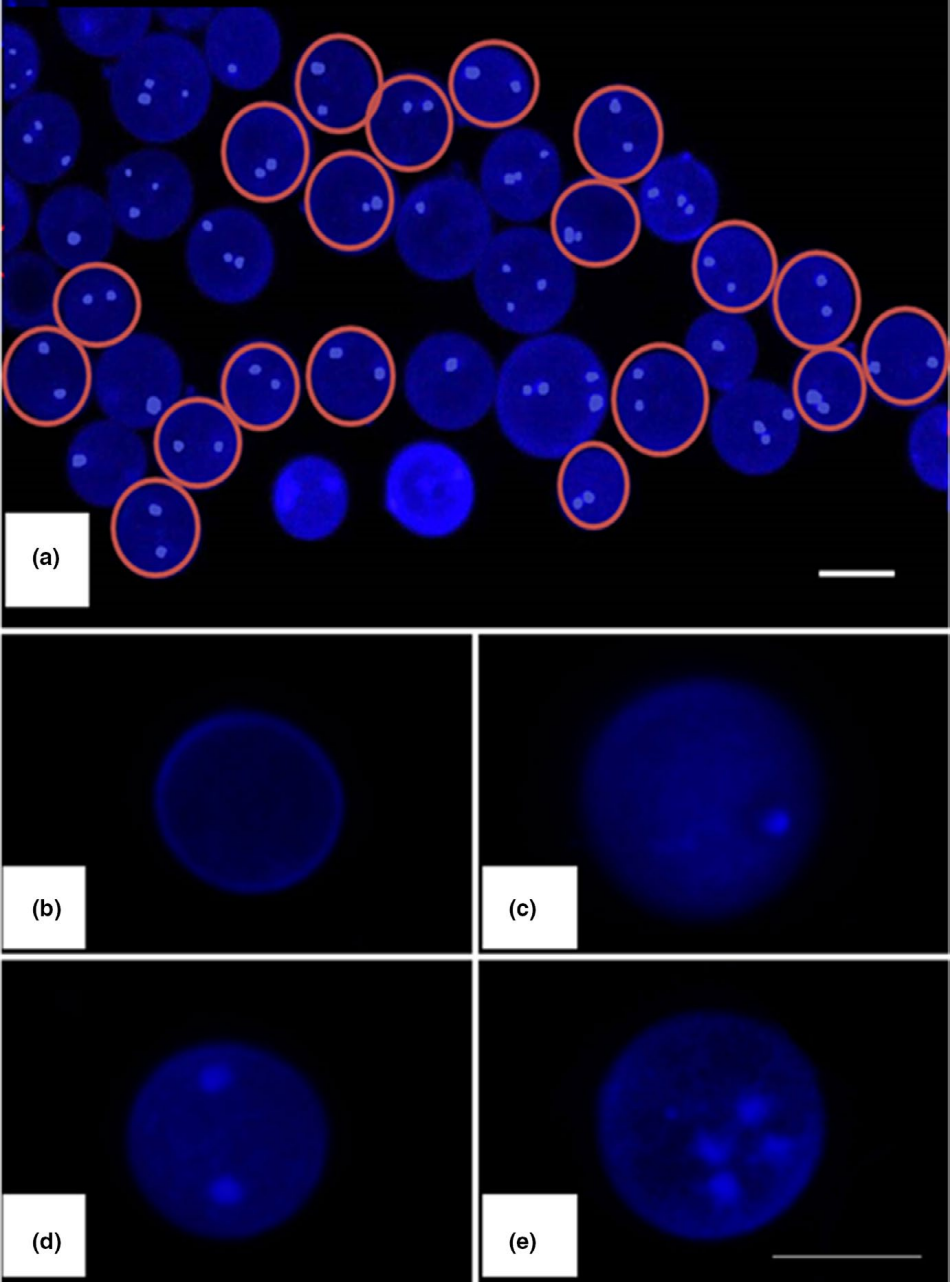
Nuclear conditions of *Oudemansiella aparlosarca* basidiospores. (a) Basidiospores observed under a fluorescence microscope. Red circles indicate binucleate spores, which were high in number; (b) Non‐nucleate spore; (c) Mononucleate spore; (d) Binucleate spore; (e) Multinucleate spore. Bars: a = 20 μm; b–e = 10 μm

**FIGURE 3:**
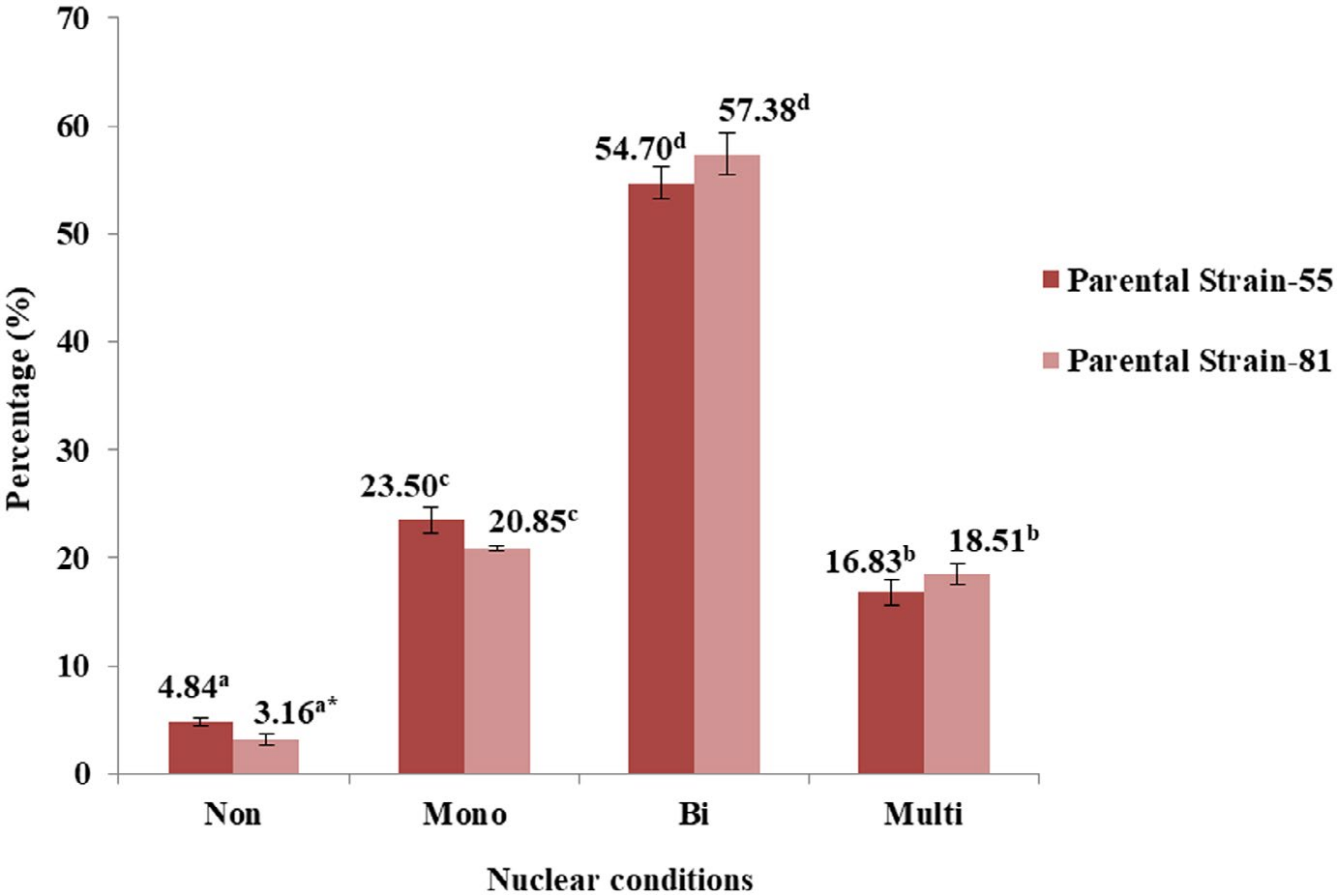
Different nuclear conditions of basidiospores of parental strains 55 and 81. Values are expressed as percentage means ± SD of five replicates, and 500 basidiospores were observed in each replicate. According to Duncan's multiple range test, means in the same‐colored bars followed by the same superscripted letter within the observed nuclear conditions in a parental strain are not significantly different at *p* < 0.05. Means in the different colored bars followed by the superscripted “*” symbol of the observed non‐nucleate condition between the parental strains are significantly different at *p* < 0.05, according to the paired‐sample *t* test. The error bars denote standard deviation

The germination percentage of basidiospores was 88%. We observed a total of 5000 basidiospores of *O*. *aparlosarca*, as shown in Figure 2a. In addition, we observed four different nuclear conditions in both parental strains (strains 55 and 81), including non‐nucleate (spore without a nucleus; Figure 2b), uninucleate/mononucleate (having one nucleus per spore; Figure 2c), binucleate (having two nuclei per spore; Figure 2d), and multinucleate (having three or more nuclei per spore; Figure 2e). Within a parental strain, all the nuclear conditions differed significantly (Figure 3). In addition, the number of binucleate spores was the highest, while the number of non‐nucleate spores was lower than that observed under the other nuclear conditions of spores in both parental strains. The percentages of binucleate, mononucleate, multinucleate, and non‐nucleate spores in strain‐55 were 54.70, 23.50, 16.83, and 4.84%, respectively, and in strain‐81, these were 57.38, 20.85, 18.51, and 3.16%, respectively. A significant difference was observed only in non‐nucleated spores, while no significant difference was observed in multi‐, mono‐, and binucleate spores between the parental strains 55 and 81 (Figure 3).

### 3.2 Spore nuclei and spore diameter

In total, we measured 4000 basidiospores. The diameters of different nuclear conditions are presented in Table 1. Non‐nucleate spores had the shortest diameter, and the average diameters of parental strains 81 and 55 were 11.52 μm and 12.15 μm, respectively. Multinucleate spores had the largest spore diameter (14.78 μm) in both parental strains. Significant differences were observed within non‐nucleate and multinucleate spores, while no significant difference was observed between the diameters of mono‐ and binucleate spores of parental strain‐55. However, in the case of parental strain‐81, significant differences were observed between the diameters of spores with all the nuclear conditions (*p* < 0.05). Although significant differences were detected in the diameters of non‐nucleate spores between the parental strains 55 and 81, no significant differences were observed in the diameters of binucleate, mononucleate, and multinucleate spores (Table 1).

**TABLE 1:** Nuclear conditions and the diameters of the basidiospores of *Oudemansiella aparlosarca* parental strain‐55 and strain‐81

Nuclear conditions	Diameter of basidiospores (μm)	
	Parental strain‐55	Parental strain‐81
Non‐nucleate	12.15 ± 0.47^a^	11.52 ± 0.30^a*^
Mononucleate	14.09 ± 0.11^b^	13.97 ± 0.10^c^
Binucleate	13.87 ± 0.32^b^	13.49 ± 0.18^b^
Multinucleate	14.78 ± 0.33^c^	14.78 ± 0.13^d^

Note

Values are expressed as percentage means ± SD of five replicates, and 200 basidiospores were observed in each replicate. Means in the same column followed by the same superscripted letter within the observed nuclear conditions in a parental strain are not significantly different at *p* < 0.05, according to Duncan's multiple range test. Means in the same row followed by the superscripted “*” symbol in the observed non‐nucleate condition between the parental strains are significantly different at *p* < 0.05, according to the paired‐sample *t* test.

### 3.3 Nuclear conditions of homokaryotic and heterokaryotic hyphal cells

Both homokaryotic and heterokaryotic strains were identified from the progeny. Among the 50 progenies isolated from each parental strain, 3 homokaryons (6%) and 47 heterokaryons (94%) were isolated from parental strain‐55 and 2 homokaryons (4%) and 48 heterokaryons (96%) were isolated from parental strain‐81. The heterokaryons were identified by observing primordia and fruiting body development.

According to our observations, the strains obtained from germinated progeny showed clamp connections after hyphal staining and were recognized as heterokaryotic strains. The strains that did not show clamp connections after hyphal staining, along with an absence of fruiting body development, were recognized as homokaryotic strains.

**FIGURE 4:**
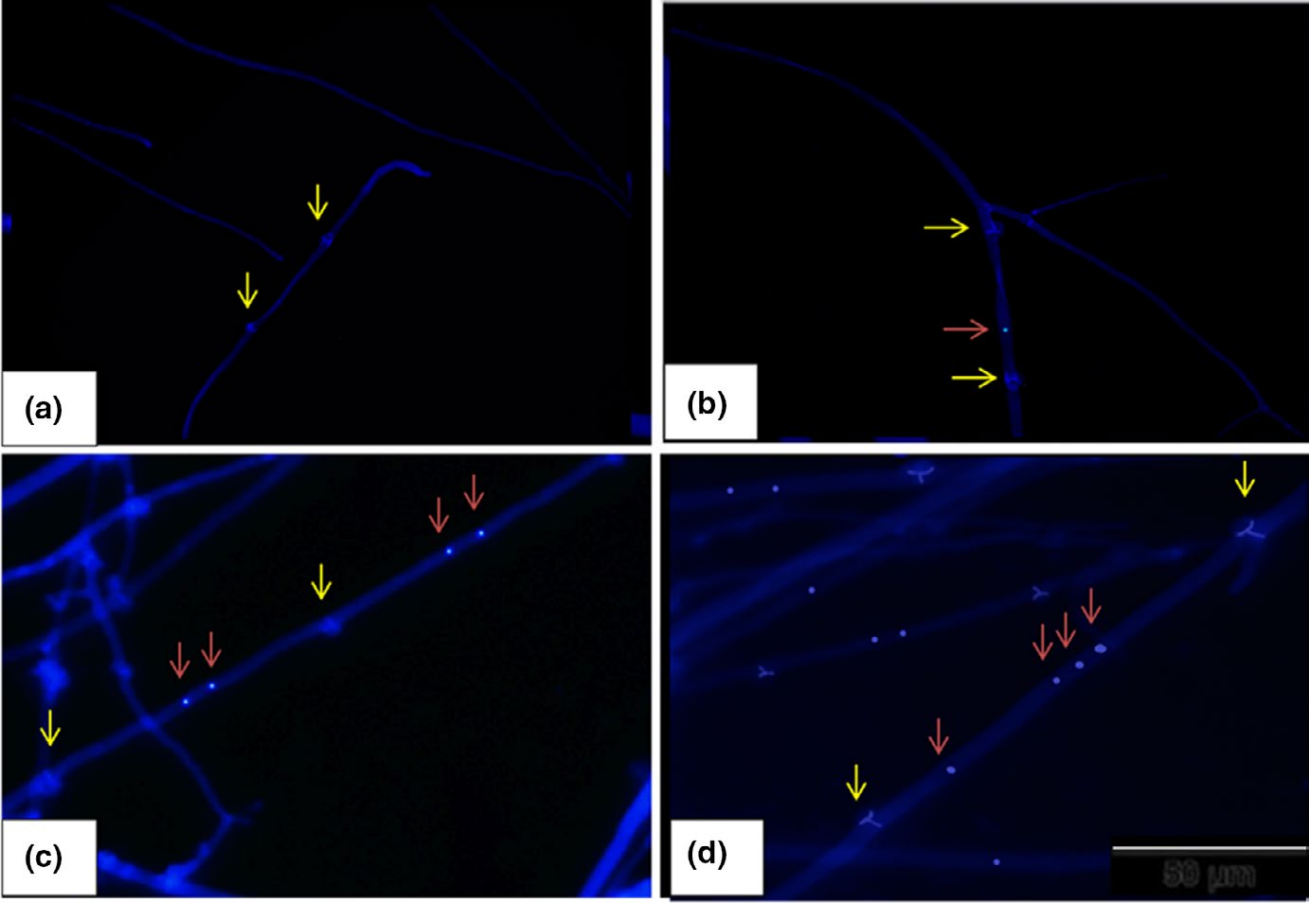
Nuclear conditions of heterokaryotic hyphal cells in the apical region of *Oudemansiella aparlosarca* strain‐55 and strain‐81, observed under a fluorescence microscope. Red arrow indicates the nucleus; yellow arrow indicates clamp connection; (a) Non‐nucleate heterokaryotic hyphal cell; (b) Mononucleate heterokaryotic hyphal cell; (c) Binucleate heterokaryotic hyphal cell; (d) Multinucleate heterokaryotic hyphal cell. Bar: 50 μm

**FIGURE 5:**
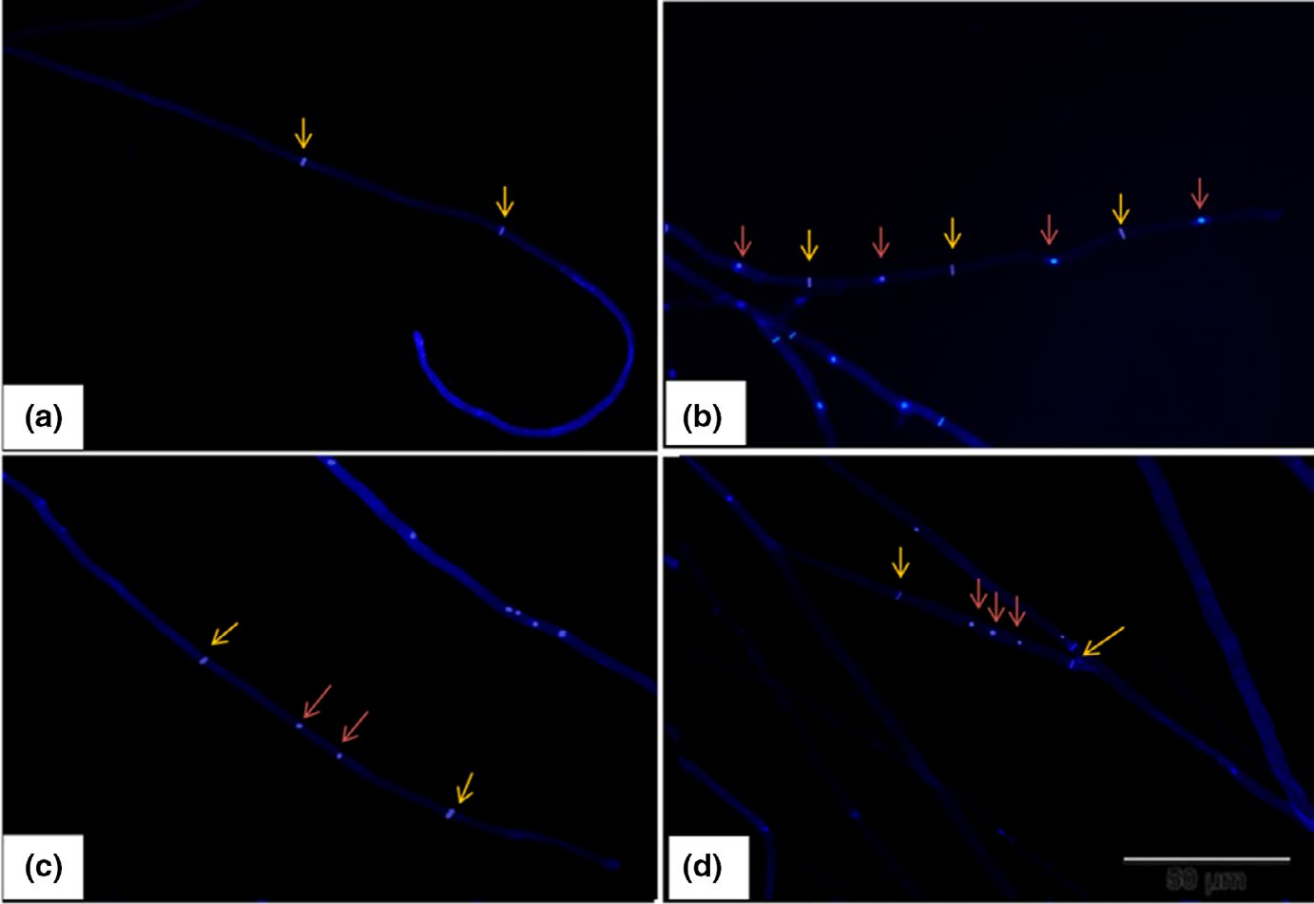
Nuclear conditions of homokaryotic hyphal cells in the apical region of *Oudemansiella aparlosarca* strain‐55 and strain‐81, observed under a fluorescence microscope. Red arrow indicates the nucleus; yellow arrow indicates septa of hyphal cell; (a) Non‐nucleate homokaryotic hyphal cell; (b) Mononucleate homokaryotic hyphal cells; (c) Binucleate homokaryotic hyphal cell; (d) Multinucleate homokaryotic hyphal cell. Bar: 50 μm

**FIGURE 6:**
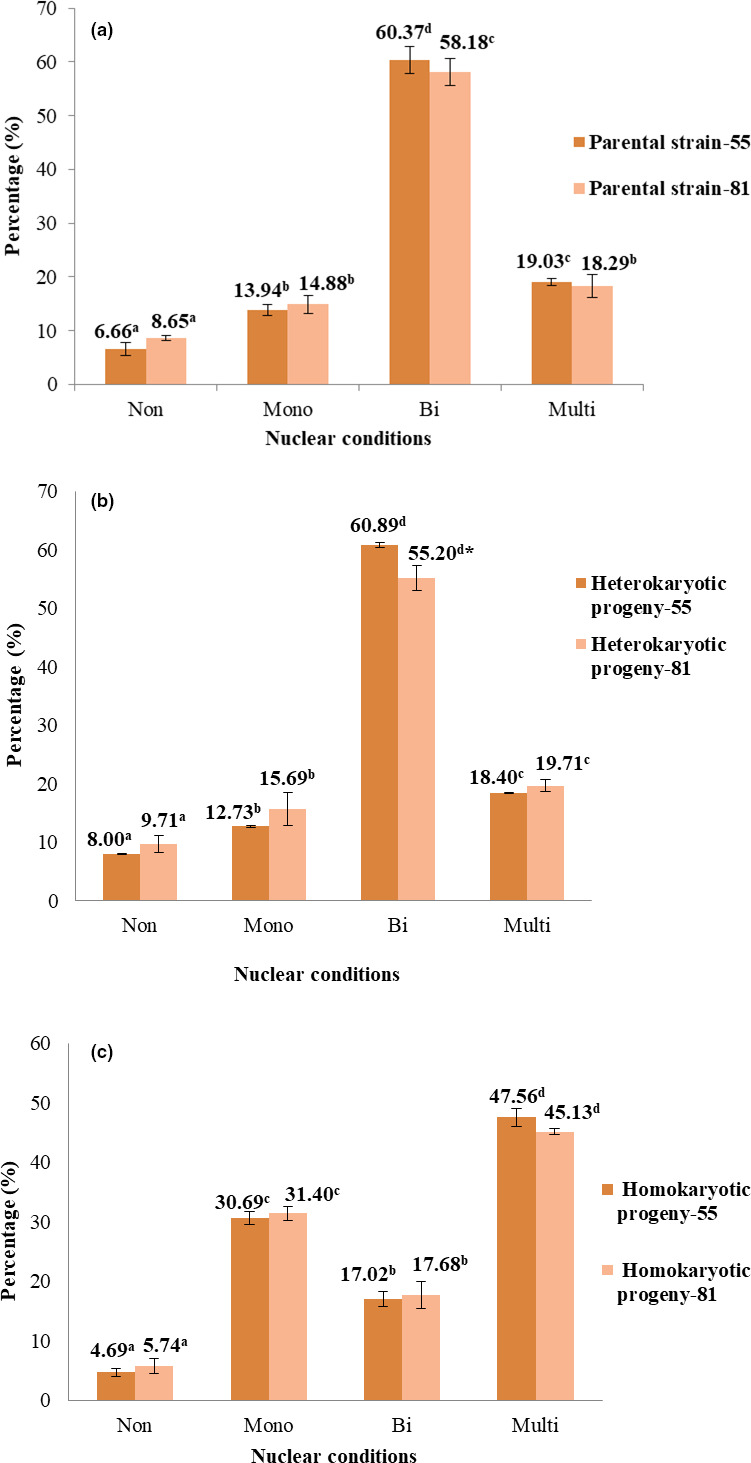
Different nuclear conditions of *Oudemansiella aparlosarca* strains 55 and 81 in heterokaryotic and homokaryotic hyphal cells. (a) Nuclear conditions of the heterokaryotic hyphal cells of parental strains; (b) Nuclear conditions of the heterokaryotic hyphal cells of the progeny; (c) Nuclear conditions of the homokaryotic hyphal cells of the progeny. Values are expressed as means ± SD of three replicates, and 50 hyphal cells in the apical region were observed in each replicate. According to Duncan's multiple range test, means in the same‐colored bars followed by the same superscripted letter within the observed nuclear conditions in a parental strain/progeny are not significantly different at *p* < 0.05. Means in the different colored bars followed by the superscripted “*” symbol of the observed binucleate condition between the heterokaryotic progenies are significantly different at *p* < 0.05, according to the paired‐sample *t* test. The error bars denote standard deviation

Similar to the nuclear conditions of the spores, both hetero‐ (Figure 4) and homo‐ (Figure 5) hyphal cells in the apical region showed four nuclear conditions in strains 55 and 81. These four configurations of nuclear conditions were irregularly distributed in the hyphal cells of a single hypha. The results of hyphal staining of experimental strains are shown in Figure 6. Similar phenomena were observed in heterokaryotic parental strains, and no significant difference was observed with respect to the nuclear conditions between the parental strains. Of note, the binucleate condition was most prominent, and the non‐nucleate condition was less prominent than the other nuclear conditions in heterokaryotic hyphal cells of both parental strains (Figure 6a). All the observed nuclear conditions of the heterokaryotic hyphal cells of the parental strain‐55 were significantly different, while only non‐nucleate and binucleate heterokaryotic hyphal cells in parental strain‐81 were significantly different (Figure 6a). In the progeny of strain‐55, binucleate hyphal cells were more numerous (60.89%) than multinucleate (18.40%) and mononucleate (12.73%) heterokaryotic hyphal cells (Figure 6b). In homokaryotic hyphal cells, 47.56% of multinucleate, 30.69% of mononucleate, and 17.02% of binucleate hyphal cells were observed (Figure 6c). These outcomes were similar to those observed in the progeny of strain‐81, wherein the percentage of binucleate (55.20%) hyphal cells was greater than that of multinucleate (19.71%) and mononucleate (15.69%) heterokaryotic hyphal cells, while multinucleate hyphal cells (45.13%) were dominant in the homokaryotic hyphal cells, followed by mono‐ (31.40%) and binucleate (17.68%) hyphal cells. The lowest percentage of non‐nucleate cells was observed in homo‐ and heterokaryotic hyphal cells of both progenies (Figure 6b,c). No significant difference was observed in the nuclear conditions of the homokaryotic hyphal cells among the progenies of strains 55 and 81. However, a significant difference was detected only in binucleate heterokaryotic hyphal cells among the progenies of strains 55 and 81. Within a progeny strain, all the nuclear conditions differed significantly (Figure 6b,c). As shown in Table 2, no significant difference was observed between the nuclear conditions of heterokaryotic parental strain‐55 and its progeny. However, the binucleate condition showed significant differences between the heterokaryotic parental strain‐81 and its progeny (Table 3).

**TABLE 2:** Nuclear conditions of the heterokaryotic hyphal cells of the *Oudemansiella aparlosarca* parental strain‐55 and respective progeny‐55

Nuclear conditions	Heterokaryotic strains‐55	
	Parental strain‐55	Progeny‐55
Non‐nucleate	6.66 ± 1.19^a^	8.00 ± 0.10^a^
Mononucleate	13.93 ± 2.49^b^	12.73 ± 0.20^b^
Binucleate	60.37 ± 0.70^d^	60.89 ± 0.47^d^
Multinucleate	19.00 ± 1.01^c^	18.4 ± 0.08^c^

Note

Values are expressed as means ± SD of three replicates, and 50 hyphal cells in the apical region were observed in each replicate. Means in the same column followed by the same superscripted letter within the observed nuclear conditions in parental strain‐55/progeny‐55 are not significantly different at *p* < 0.05, according to Duncan's multiple range tests.

**TABLE 3:** Nuclear conditions of the heterokaryotic hyphal cells of the *Oudemansiella aparlosarca* parental strain‐81 and respective progeny‐81

Nuclear conditions	Heterokaryotic strains‐81	
	Parental strain‐81	Progeny‐81
Non‐nucleate	8.65 ± 0.40^a^	9.71 ± 1.45^a^
Mononucleate	14.88 ± 2.51^b^	15.69 ± 2.86^b^
Binucleate	58.18 ± 2.08^c^	55.2 ± 2.10^d*^
Multinucleate	18.29 ± 1.72^b^	19.71 ± 1.05^c^

Note

Values are expressed as means ± SD of three replicates, and 50 hyphal cells in the apical region were observed in each replicate. Means in the same column followed by the same superscripted letter within the observed nuclear conditions in parental strain‐81/progeny‐81 are not significantly different at *p* < 0.05, according to Duncan's multiple range tests. Means in the same row followed by the superscripted “*” symbol in the observed binucleate condition among the parental strain‐81 and progeny‐81 are significantly different at *p* < 0.05, according to paired‐sample *t* test.

## 4 DISCUSSION

In this study, we investigated the different nuclear conditions of the basidiospores of *O*. *aparlosarca* and determined their diameters. In addition, we examined the different nuclear conditions of homokaryotic and heterokaryotic hyphal cells in the apical region of *O*. *aparlosarca*. Four nuclear conditions, namely, non‐nucleate, mononucleate, binucleate, and multinucleate, were observed in the basidiospores and homokaryotic and heterokaryotic hyphal cells. Although there were some phenotypic differences between the two parental strains, their sequences were highly similar (around 97%‒99%) to the original sequence of *O*. *canarii* in the NCBI, suggesting they belong to the same species.

Basidiospores typically contain a single haploid nucleus, and on germination, they produce homokaryotic hyphae with one nucleus per hyphal cell (Nieuwenhuis et al., 2013). Cell fusion between two sexually compatible homokaryotic hyphae leads to the formation of heterokaryotic hyphae, which contain two haploid nuclei with a clamp connection joining two contiguous hyphal cells (Gladfelter & Berman, 2009). Under ideal climatic conditions, the heterokaryotic primordia develop into mushrooms, and the mature fruiting body develops into mononucleate, haploid basidiospores to continue its life cycle (Miles & Chang, 2004). The present study revealed that binucleate basidiospores were dominant followed by mono‐, multi‐ and non‐nucleate basidiospores, with significant differences. This observation was simultaneously confirmed in two isolates of *O*. *aparlosarca* (parental strains 55 and 81).

Meiosis is followed by post‐meiotic mitosis in secondary homothallism, which occurs in the top portion of the basidium in basidiomycetes. Occasionally, this occurs inside the sterigmata or pre‐mature spore. In pattern D, as described by Duncan and Galbraith (1972), post‐meiotic mitosis occurs inside basidiospores and both nuclei persist inside the spores, which are called binucleate spores. In pattern F, post‐meiotic mitosis occurs inside the basidium, and two nuclei migrate from the basidium to each spore (Mueller et al., 1993), increasing the number of nuclei in the spores. Additionally, some spores do not develop properly and/or do not receive any post‐meiotic nucleus (Ling et al., 2019). In the present study, the occurrence of post‐meiotic mitosis might have led to the formation of heterokaryotic or homokaryotic binucleate spores.

In *A*. *bisporus*, homokaryotic spores are generally produced in the heterothallic lifecycle, while heterokaryotic spores are vastly produced in the amphithallic life cycle, often referred to as the secondary homothallic life cycle (Callac et al., 1993; Kamzolkina et al., 2006). Previous studies on ectomycorrhizal basidiomycetes have reported that *Amanita*, *Cortinarius*, *Hebeloma*, *Inocybe*, *Laccaria*, and *Suillus* genera produce binucleate basidiospores (Horton, 2006). Although *A*. *bitorquis* has binucleate heterokaryons, most *Agaricus* species possess multinucleate heterokaryons lacking clamp connections (Ling et al., 2019; Raper, 1976; Raper et al., 1972). However, Souza Dias et al. (2008) reported that binucleate spores that are functionally similar to mononuclear homokaryons are present in *A*. *brasiliensis*.

The results of the present study demonstrated that approximately 71%–75% of basidiospores were binucleate and multinucleate, and on examination of progeny, we found that only 4%–6% were homokaryons and 94%–96% were heterokaryons. Since both homokaryotic and heterokaryotic progeny were identified, we presumed that *O*. *aparlosarca* showed an amphithallic life cycle along with post‐meiotic mitosis. We speculated that the predominantly observed binucleate and multinucleate spores might form heterokaryotic progeny.

Asynchronous nuclear migrations, which can lead to unequal distribution of nuclei in spores, have been observed in *Pisolithus microcarpus* from basidium to basidiospores (Campos & Costa, 2010). Such occurrence also might have led to the formation of multinucleate basidiospores observed in the present study. A few non‐nucleate basidiospores may be produced when they do not receive any post‐meiotic nucleus. Here, we observed a spore germination rate of 88%. Of note, the non‐nucleate condition in basidiospores was around 5%, which may explain, in part, the 12% of non‐germinated spores. Further, there are chances that, due to handling errors during manual picking and placement of the isolate spores using fine needles, damage to the spores may have occurred. Further detailed studies on basidia and basidiosporogenesis will facilitate a thorough understanding of the physiology and lifecycle of *O*. *aparlosarca*.

A significant difference was observed only in the diameters of non‐nucleate basidiospores among the parental strains 55 and 81. In general, the largest diameter was observed for multinucleate spores, and the smallest diameter was observed for non‐nucleate spores of the parental strains. As the number of nuclei increased, the diameters of basidiospores also increased in parental strain‐81. However, a similar increase in diameter was not observed in the mono‐ and binucleate spores of parental strain‐55. The size of basidiospores depends on several factors, such as nutritional mode (saprotrophic/facultative/parasitic), distribution, spore shape, basidiocarp size, and resource (conifer/deciduous/both; Kauserud et al., 2008). The basidiospores observed in the present study showed thick cell walls (around 3 μm), which helps in protecting the spores. The diameter of the spores was not affected by the temperature–time combination used in the heating‐cooling cycles previously described in the spore staining method. Generally, spore size is universally used to determine fungal taxonomy. However, basidiospores with different nuclear conditions and different diameters can be used for genetics and breeding studies on *O*. *aparlosarca* to determine the percentage of spores with different nuclear conditions that give rise to homokaryotic vs. heterokaryotic mycelium, to avoid the selection of non‐nucleate spores in single spore isolation during the breeding process, and to gain a deep understanding of basidiosporogenesis.

Traditionally, light microscopic observations have been used to distinguish heterokaryotic hyphae from homokaryotic hyphae through clamp connections. The studied strains possessed dense hyphae with several layers, thereby making it difficult to distinguish between them based on clamp connections. Therefore, we used the hyphal cell staining method for easy identification of heterokaryons from homokaryons. A large number of binucleate cells were observed in the heterokaryotic hyphal cells of the parent and their progeny, and multinucleate cells were more common in the homokaryotic hyphal cells of the progeny than in other nuclear conditions. The non‐nucleated condition was the least dominant in both homokaryotic and heterokaryotic hyphal cells of the parent and progeny.

The presence of mono‐, bi‐, and multinucleate hyphal cells has also been reported in *Rhizopogon roseolus*. However, binucleate cells were the most common in both homokaryotic (more than 70%) and heterokaryotic (around 86%) hyphal cells (Sawada et al., 2014). The results of the present study demonstrated that around 60% of hyphal cells contained binucleate heterokaryotic hyphae. In addition, multinucleate hyphal cells (around 20%) and mononucleate and non‐nucleated (around 20%) hyphal cells were observed with clamp connections in the apical region of heterokaryotic hyphae. In homokaryotic hyphal cells, around 40% of the multinucleated cells were mainly present in the progeny compared with that in other nuclear conditions. Our findings indicate that even though clamp connections can be used to separate heterokaryons from homokaryons, the nuclear condition of hyphal cells is not always a key characteristic that can be used to distinguish between homokaryotic hyphae and heterokaryotic hyphae. Additionally, our results were similar to those of a study on *Lentinula edodes*, which showed that around 52% of the binucleate heterokaryotic hyphal cells and around 41% of the multinucleate homokaryotic hyphal cells were present in the initial stage of culture (Gao et al., 2019).

## 5 CONCLUSION

The amphithallic life cycle of *O*. *aparlosarca* includes homokaryotic and heterokaryotic basidiospores. The predominance of binucleate spores might be a result of post‐meiotic mitosis. Different nuclear conditions influence the diameter of basidiospores. The diameters of basidiospores can be used to distinguish mono‐ and binucleate spores from non‐ and multinucleate spores in *O*. *aparlosarca*. The hyphal staining method was an important method to observe clamp connections in the heterokaryons in our study. Heterokaryotic hyphal cells were predominantly binucleate cells, while homokaryotic hyphal cells were mostly multinucleate cells.

## CONFLICT OF INTEREST

None declared.

## AUTHOR CONTRIBUTIONS

**Roy Rebecca:** Conceptualization (equal); Data curation (lead); Investigation (equal); Methodology (lead); Software (equal); Validation (lead); Writing‐original draft (equal); Writing‐review & editing (equal). **Qi Gao:** Conceptualization (lead); Data curation (supporting); Methodology (supporting); Resources (equal); Validation (lead); Writing‐original draft (equal); Writing‐review & editing (equal). **Yujin Cui:** Data curation (supporting); Investigation (supporting); Methodology (supporting); Resources (lead); Software (equal). **Chengbo Rong:** Data curation (supporting); Investigation (equal); Methodology (supporting); Resources (equal); Visualization (supporting). **Yu Liu:** Conceptualization (equal); Funding acquisition (equal); Methodology (supporting); Project administration (equal); Visualization (supporting). **Wensheng Zhao:** Conceptualization (equal); Methodology (equal); Project administration (equal); Resources (supporting); Visualization (equal). **Wasantha Kumara:** Conceptualization (equal); Project administration (equal); Resources (supporting); Supervision (equal); Visualization (equal). **Shouxian Wang:** Conceptualization (equal); Funding acquisition (equal); Project administration (equal); Resources (equal); Supervision (lead).

## ETHICS STATEMENT

None required.

## Data Availability

The data generated or analyzed during this study have been provided in this article. The sequence data of *O*. *canarii* (internal transcribed spacers and rRNA) are available in the NCBI database under the accession number MK336783: https://www.ncbi.nlm.nih.gov/nuccore/MK336783.
